# Recovering the raw data behind a non-parametric survival curve

**DOI:** 10.1186/2046-4053-3-151

**Published:** 2014-12-30

**Authors:** Zhihui Liu, Benjamin Rich, James A Hanley

**Affiliations:** Dalla Lana School of Public Health, University of Toronto, 155 College St, M5T 3M7 Toronto, Canada; Cancer Care Ontario, 505 University Avenue, M5G 1X3 Toronto, Canada; McGill University Health Centre, 687 Pine Avenue West, H3A 1A1 Montreal, Canada; Department of Epidemiology, Biostatistics and Occupational Health, McGill University, 1020 Pine Avenue West, H3A 1A2 Montreal, Canada

**Keywords:** Data recovery, Survival curves, PostScript, Digitization, Accuracy

## Abstract

**Background:**

Researchers often wish to carry out additional calculations or analyses using the survival data from one or more studies of other authors. When it is not possible to obtain the raw data directly, reconstruction techniques provide a valuable alternative. Several authors have proposed methods/tools for extracting data from such curves using a digitizing software. Instead of using a digitizer to read in the coordinates from a raster image, we propose directly reading in the lines of the PostScript file of a vector image.

**Methods:**

Using examples, and a formal error analysis, we illustrate the extent to which, with what accuracy and precision, and in what circumstances, this information can be recovered from the various electronic formats in which such curves are published. We focus on the additional precision, and elimination of observer variation, achieved by using vector-based formats rendered by PostScript, rather than the lower resolution image-based formats that have been analyzed up to now. We provide some R code to process these.

**Results:**

If the raster-based images are available, one can reliably recover much of the original information that seems to be ‘hidden’ beneath published survival curves. If the original images can be obtained as a PostScript file, the data recovered from it can then be either input into these tools or processed directly. We found that the PostScript used by Stata discloses considerably more of the data hidden behind survival curves than that generated by other statistical packages.

**Conclusions:**

When it is not possible to obtain the raw data from the authors, reconstruction techniques are a valuable alternative. Compared with previous approaches, one advantage of ours is that there is no observer variation: there is no need to repeat the digitization process, since the extraction is completely replicable.

## Background

Researchers often wish to carry out additional calculations or analyses using the survival data from studies of other authors. Since it is not always possible to obtain the raw data directly from the authors, one is forced to make do with the information that can be recovered from the articles. The researchers differ in their reasons for obtaining such data, and in the number of studies involved. Our own experiences [1–3] focus on randomized trials of cancer screening, where the mortality deficits produced by cancer screening are delayed. Thus, a sequence of time-specific hazard ratios (i.e., a rate ratio ‘curve’) that accommodates this delay is more appropriate than the single-number hazard ratio typically reported by trialists. However, our methodology is applicable to any situation where published data are in the form of cumulative incidence curves, or survival curves, of a step function form.

Some guidance on data reconstruction can be found in the meta-analysis literature, since the summaries are not always reported in the way meta-analysts would wish and since simplifying assumptions, such as a constant hazard ratio, may be inappropriate [4–6]. Duchateau [7] expressed caution, pointing out that the number of events should not be estimated from the Kaplan-Meier curves for meta-analytic purposes unless virtually no patients are lost to follow-up or censored and there are still many patients at risk in the two groups at the time at which the number of events is to be determined. Other authors have shown that in some circumstances, and by making some assumptions, it is possible to extract additional information. Among the earliest to do so were Parmar [8], who described how to estimate the log of the hazard ratio, and its variance, from the survival curves themselves, rather than from numbers and summaries reported in the text. Although their focus was on assessing the accuracies of different techniques for combining published survival curves, Earle [9] et al. are one of the first to mention using digitized images, obtained by ‘scanning the survival curves and imported them into the CorelDRAW! 3.0 graphics package.’ They, and several others since then, have focused on the many practical challenges: Williamson [10] illustrated how information on the numbers at risk may be used to improve the estimation; Tudur [11] reviewed the practicality and value of previously proposed methods; Tierney [12] provided a spreadsheet to estimate hazard ratios and associated statistics from published summary statistics or data extracted from Kaplan-Meier curves. The grImport package is intended to add extracted images to R plots, but in the ‘Scraping data from images’ section in [13], Murrell extracts data from a survival curve and shows that the resulting curve matches the original.

Most recently, Guyot et al. [4] provide a method (and R code) to ‘derive from the published Kaplan Meier survival curves a close approximation to the original individual patient time-to-event data from which they were generated.’ They did so ‘using an algorithm that maps from digitized curves back to KM data by finding numerical solutions to the inverted KM equations, using where available information on number of events and numbers at risk.’ They assessed the reproducibility and accuracy of several statistics based on reconstructed KM data by comparing published statistics with statistics based on repeated reconstructions by multiple observers.

Increasingly, the figures in electronic publications are *vector*-based and rendered by PostScript, rather than *image*-based. Thus, in this note, we take advantage of this much higher resolution to eliminate the variation introduced by human digitizers and achieve greater precision and accuracy. The much greater precision can also be used to gain greater detail as to numbers at risk at various time points, and the approach can handle survival curves containing hundreds of steps.

Using worked examples and a formal error analysis, we illustrate the extent to which, and with what accuracy and precision, and in what circumstances, the original information can be recovered from the vector-based and image-based formats in which such curves are published. We describe an R function we use to extract the relevant PostScript data used to draw lines and to convert the PostScript co-ordinates to co-ordinates in the time-survival {*t*,*S*(*t*)} space. If users wish, these can then be used as input to the R software provided by Guyot et al. [4], or the spreadsheet provided by Tierney [12], or further processed directly by the user. Our own applications have been in estimating yearly event rates using aggregated person time and event counts, rather than in reconstructing individual-level data, but what we describe can be applied to both.

In some instances, it is possible to obtain even more than was visible to the human eye, or a digitizer, and we describe a Stata-specific data disclosure practice that helped in that respect. Before doing so, we first briefly review the general principles that one can use to derive as much information as possible from a non-parametric survival curve.

## Methods

### Principles

To start with, we will assume that the Kaplan-Meier or Nelson-Aalen curve values can be measured with sufficient accuracy and precision (we will relax this requirement in later sections). In such cases, first principles - and some deductions - generally allow one to recover not only (i) the distinct ‘event’ time *t* that defines each risk set [we denote the ordered distinct event times by *t*_1_,*t*_2_,…,*t*_*k*_] but also for each risk set (ii) the number at risk *n* and (iii) the number of events *d*. Then, by successive subtractions, one can calculate (iv) the number of observations censored between successive risk sets *c*. Unless the exact times of censored observations are indicated on the graph, the recovered data can be compressed into the sequence


If the exact censoring times are indicated on the graph, then in principle, the entire dataset can be reconstructed; otherwise, the best that one can do is to use interpolation, together with the description of the recruitment period and closing dates of the study, to impute the locations of the censored observations within the various time intervals. Most authors have spaced them uniformly within these intervals.

**Figure 1:**
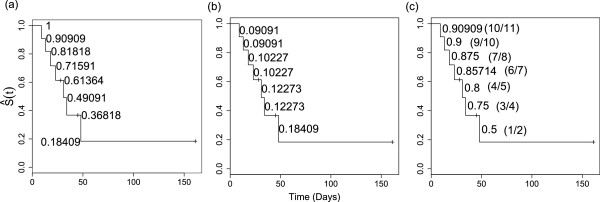
**Kaplan-Meier survivor function, showing the heights, jumps, and ratios of heights.** **(a)** Kaplan-Meier estimate of the survivor function for patients with AML in the maintained group, showing the heights *S*(*t*_*j*_). **(b)** Same K-M curve showing the jumps *J*(*t*_*j*_). **(c)** Same K-M curve showing the ratios of heights *S*(*t*_*j*_)/*S*(*t*_*j*−1_). The curve shown in each panel was fitted and drawn using the survival package in R.

To review the principles and illustrate the reasoning, we begin with a small example, using a widely used illustrative dataset. Figure 1a shows the Kaplan-Meier estimate of the survivor function for patients with acute myelogenous leukemia (AML) in the ‘maintained’ group, available in the survival package [14] in R. The question at the time was whether the standard course of chemotherapy should be maintained for additional cycles for these patients. To start with, we ask the reader to ignore the additional information we show on each panel and to limit their attention to the curve, with its steps and censoring marks.

Let *S*(*t*_*j*_) denote the survival probability, or the ‘height’ of the survival curve, at time *t*_*j*_ and define the ‘jump’ *J*(*t*_*j*_) as *S*(*t*_*j*−1_)−*S*(*t*_*j*_). We usually would know it, but suppose we do not even know *n*_0_, the number of subjects at time *t*_0_=0. Without any other information except the step function values and the times of the steps, how much of the raw information can one recover from such a graph, if the *S*’s are known with sufficient accuracy? (By sufficient accuracy, we mean that the true value can be reliably deduced to be *n*_*j*_ and not *n*_*j*_−1 or *n*_*j*_+1).

A quick inspection of Figure 1a shows that there are seven jumps and three censoring marks, so *n*_0_ is at least 10. Even *without* censoring marks, the differences in the size of the jumps indicate *some* censoring - if there were none, all jumps would be either of equal size (1/*n*_0_), or multiples of this, i.e., *m*/*n*_0_ if *m*>1 events in a risk set. As shown in Figure 1b, *J*(*t*_3_)>*J*(*t*_2_), while *J*(*t*_5_)>*J*(*t*_4_), and *J*(*t*_7_)>*J*(*t*_6_); in addition, since the last observation is censored, we can infer that there must be at least four censored values in total.

One way to understand why (single-event) jumps located further to the right can *only be larger* than those that precede them is via Efron’s re-distribution-to-the-right algorithm [15]: initially, a probability mass of 1/*n*_0_ is placed at each observation time. Proceeding from left to right, as a censored time is encountered, its mass is redistributed in equal portions to all observations on its right. This procedure of sweeping out the censored observations is repeated until all of their associated masses have been redistributed.

In Figure 1b, the first two jumps *J*(*t*_1_) and *J*(*t*_2_) are of equal size of 0.09091, or 1/11, suggesting that there may have been initially 11 persons at risk (of course, without having further information, it could also have been 22 or 33, but subsequent values of the curve will effectively rule these out). The fact that the third jump is bigger establishes that there must be a censored observation at or after *t*_2_ and before *t*_3_. But since (unlike the other censored observations that fall strictly between events times) it is not denoted by a tick mark on the graph, the censoring must, by convention, have occurred *immediately after* the event(s) at *t*_2_, but due to the discreteness of the data, have been recorded as a ‘ *t*_2_+’. Thus, while censoring marks may give more precise locations of the censored observations, statistical packages do not necessarily display *all* of them, and so one should not rely on identifying all of them just from the tick marks.

Following Efron’s algorithm, *J*(*t*_3_) of size 0.10227 can be seen to be the sum of the original mass of 1/11 (0.09091) and (1/8)th of the same size mass associated with the censored ‘ *t*_2_+’ observation that was redistributed among the eight who were at risk just after *t*_2_, i.e., *J*(*t*_3_)=*J*(*t*_2_)+1/8×*J*(*t*_2_). However, the arithmetic and the multiple possible ‘legacies’ and configurations become complicated, if there are multiple events at the same observed time, or if more than one observation in an interval is censored. Thus, as the expressions for absolute sizes of the jumps start to become complicated, how else might we determine the numbers at risk - and the numbers of events - at the time of each successive jump?

We found it easiest to first assume that each *d*_*j*_=1, then derive the corresponding *n*_*j*_, then use any anomalies in the pattern of successive *n*_*j*_s to revise *d*_*j*_ to a larger integer, and scale the corresponding *n*_*j*_ down accordingly. One way to go from *d*_*j*_ to *n*_*j*_ is to exploit the ‘product of conditional survival probabilities’ structure of the K-M estimator: reverse the sequence of products that are used as the estimator and divide the  by . The resulting ratio is 1−*d*(*t*_*j*_)/*n*(*t*_*j*_), where *d*(*t*_*j*_) denotes the number of events at time *t*_*j*_ and *n*(*t*_*j*_) is the number at risk at time *t*_*j*_. If we can establish what *d*(*t*_*j*_) is, then we get the *simple expression* for *n*_*j*_:
1

Indeed, as shown in Figure 1c, we can infer by using this expression that the numbers at risk at {*t*_1_,…,*t*_7_} are {*n*_1_,…,*n*_7_}={11,10,8,7,5,4,2}.

**Figure 2:**
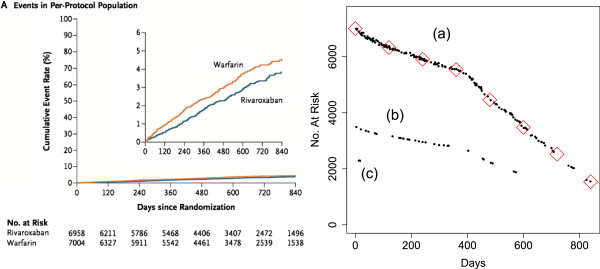
**Cumulative event rates and estimated numbers at risk.** (Left) Cumulative event rates in atrial fibrillation patients who received warfarin or rivaroxaban. (Right) The vertical location of each dot represents the estimated number at risk in the warfarin arm in the risk set in question (horizontal location). The numbers were derived by applying Equation 1 to the *S*(*t*_*j*_) estimates derived from the PostScript commands used to render the vector image. The diamonds represent numbers at risk at days 0, (120), 840, reported at the bottom of the figure in the article. Clearly, even if they had not been provided, they could have been very accurately estimated just from the successive *S*(*t*_*j*_) estimates alone. The slight lack of monotonicity in series **(a)** reflects rounding errors in the PostScript co-ordinates. Each *n*_*j*_ in series **(b)** is based on the (clearly false) assumption that the corresponding *d*_*j*_=1; at these distinct failure times, clearly, *d*_*j*_=2, so each *n*_*j*_ is twice that shown. Likewise, the *n*_*j*_s in series **(c)** are based on assuming *d*_*j*_=1, when, again clearly, *d*_*j*_=3, and the *n*_*j*_ should be three times that shown.

The initial numbers - which are usually reported in publications - and the sequence of ‘fitted’ or ‘inferred’ numbers at risk, can be used to establish with virtual certainty *the number of events at each distinct event time - thed*_*j*_*s*. If there indeed is a single event at each distinct event time, then the inferred numbers at risk will - apart from the (usually small) measurement errors - form a monotonically decreasing sequence. Systematic departures from monotonicity are immediately evident: if there were in fact two events at a distinct event time, the ‘fitted’ number at risk, *n*_*j*_, will be 1/2 of what it should be, and will stand out distinctly from its singleton-based neighbors; if there were three events, the ‘fitted’ number at risk will be 1/3 of its neighbors, and so on. We will illustrate this later when discussing the example in Figure 2 (right). From the {*s*_1_,…,*s*_7_} thus established, and the {*n*_1_,…,*n*_7_}, we can then by subtraction deduce that in our example {*c*_1_,…,*c*_7_}={0,1,0,1,0,1,1}.

If the time spacings between the adjacent *t*s are relatively short, or if the numbers at risk at specific time points (e.g,. yearly or monthly) are indicated on the graph, then by further interpolation of the sequence of numbers at risk, the total amounts of person time for each time interval of interest can be established with minimal error. Survival plots typically have a width:height aspect ratio larger than 1. Thus, the relative errors will tend to be smaller on the ‘time’ than on the ‘person’ dimension of the person-time denominator inputs to the calculated event rates.

The above formula referred to the Kaplan-Meier curve. If instead of the survival curve, the graph shows the Nelson-Aalen estimator of the *cumulative hazard rate function*, given by , then the expression for *n*(*t*_*j*_) is
2

It is not always obvious from the label the vertical axis whether an increasing ‘Nelson-Aalen’ curve refers to this sequence of *H*s, i.e., integrated hazards, or to the cumulative incidence, or risk, i.e., CI_*j*_=*R*_*j*_=1− exp[−*H*_*j*_]. If indeed it is the latter, i.e., the complement of *S*, then the formula for *n*_*j*_ becomes
3

Until now, we have assumed that the vertical and horizontal co-ordinates of the vertices can be measured with ‘sufficient’ accuracy. We now turn to what can be achieved using the actual K-M and N-A curves that can be extracted from bitmap images and vector-based graphics in publications.

### Practicalities

Just a decade or two ago, it was still common, but time-consuming, to use of the ‘pencil and ruler’ approach to ‘read off survival probabilities’ [8] from a (possibly enlarged) hardcopy graph. This practice could involve substantial measurement error, especially when the print was small or the resolution was poor. Today, since most graphs can be either accessed electronically or converted into such a format, the labor intensive work can be reduced, with improved precision and accuracy. In our website http://www.med.mcgill.ca/epidemiology/hanley/software/DataRecovery, we have collected together a number of graphs found in electronically published articles. Those images are typically of two types, what the Adobe Acrobat documentation refers to as ‘raster images’ and ‘vector objects’.

#### Raster images

A raster image, or bitmap, consists of pixels (the smallest addressable screen elements in a display device) arranged in a two-dimensional grid. Each pixel, represented by a dot or square, has its own coordinates and color. When one zooms in more and more, the image becomes grainier and the individual dots that make up the lines and symbols on the graph become more evident.

In a black and white or grayscale image, white is typically represented by the value 1, black by a 0, and gray by an intermediate value; color images use a more elaborate coding scheme involving multiple channels, such as RGB or CMYK. Just as in digital photography, the larger the numbers of pixels, the more faithful the representation of the original values. For an example from prostate cancer screening (a topic to be discussed further below), see Figures Two and Three in the article by Andriole [16].

Raster images can be stored in a number of file formats; the most common are.jpeg,.png,.tiff, and.gif. They can be generated in a number of ways, such as (i) scanning the hardcopy and storing it as a raster image, (ii) (if it is in a page of an electronic document) zooming in on the area containing the graph and taking a screenshot, or (iii) (if it is already embedded in a PDF file) using the ‘export images’ feature in Adobe Acrobat.

The desired points on the graph can be extracted from the image file in one of two ways. The more technical way is to use a programming language such as Basic, C++, or SAS to read the color values into a 2-D array, identify from the colors of the dots the pixel locations of key landmarks (such as the axes intersect, and the furthest apart vertical and horizontal tick marks), and finally determine which sequences of pixel locations contain the dots that make up the curves of interest. Whereas the ReadImages package [17] makes it easy to read the array into R, the programming to process the array is still a considerable challenge, particularly for the portions where curves overlap.

The easier way is to use a graph digitizer, a computer program which (i) imports and displays the selected image on the screen and (ii) allows the user to identify horizontal and vertical landmarks by way of the cursor and to click on as many locations on the graph as are desired, then converts and stores the corresponding (*x*,*y*) values. A number of graph digitizers (such as *GraphClick*, *Engauge Digitizer* and *Plot Digitizer*) are available for free on the web. Guyot et al. [4] report that the software *DigitizeIt* (http://www.digitizeit.de/) performed well. Because digitizations of raster images have been covered in detail by Guyot et al. [4], we will not give examples but merely contrast their accuracy with those of vector images in the theoretical error analysis below.

#### Vector images

A vector-based figure or graph consists of geometrical primitives or elements such as points and lines; it can be identified by the fact that it can be enlarged indefinitely without loss of quality. Two endpoints of a line are represented by two (*x*,*y*) pairs and a dot by a line of zero length. The ‘Post’ in PostScript - the most common language for producing them - refers to the principle of device independence: the elements are rendered in real time from the stored co-ordinates of the elements, regardless of the local hardware on which the software is used. This portability principle underlies the portable document format (PDF), developed by Adobe; PDF files are based on the PostScript language.

The contents of a PDF document are typically stored as a binary file, but both the Adobe Acrobat Pro application, and the Preview application provided in Mac OS, can export a PDF document (or the page of it that contains the graph of interest) as a PostScript file, which contains the commands. Such files tend to be large and contain much technical information, but it is easy (if tedious) to identify the commands that produce the axes, tick marks, and the sequence of line segments or dots that make up the K-M and N-A curves.

In PostScript, locations on a page are measured in printer points (72 points per inch) from the upper left corner of the page. Thus, a 2 inch (144 point) *x*-axis, extending from *t*=0 and *t*=5, and physically from 1 to 3 in from the left side of the page and located 5 in (360 points) below the top of the page would be specified by the line segment (72, 360) ⇔ (216, 360). Suppose that the ends of the 1.5-in (108 points) high *y*-axis correspond to *S*=0 and *S*=1, respectively. Then, from these PostScript co-ordinates, we can determine that the line segment (144, 300) ⇔ (146.88, 300) is a horizontal portion of the step function taking the value *S*=(360−300)/108=0.555 in the interval *t*=(144−72)/(144/5)=2.5 to *t*=(146.88−72)/(144/5)=2.6 and that the segment (146.88, 300) ⇔ (146.88, 303) is a vertical jump at *t*=2.6, of length *ΔS*=3/108=0.028 from *S*=0.555 to *S*=0.583.

Surprisingly, some publications include a mix of formats. Indeed, in the publication used as the source of Figure One of [4], the axes in the original New England Journal of Medicine (NEJM) figure had been rendered as vectors in PostScript, but the two curves are superimposed as an image. The composite was analyzed as an image by Guyot et al. [4]. By contrast, the other figure in that NEJM publication was rendered entirely in PostScript, albeit with some very complex paths to form the line segments.

### Precision

How precise are the data extracted from raster and vector images? One can assess this question at a number of levels, beginning with the precision of the (or ) measurements themselves. Consider a typical 300 dots per inch (dpi) *raster* image in which the full (0, 1) *S*-axis is 1.6 in, or 480 pixels, high. This gives a resolution of *ΔS*≈0.002. (A ‘downwards’ curve that ends at say *S*=0.9, but on a plot that uses the full (0,1) scale, squanders considerable precision: it makes more sense to plot the ‘upwards’ function, 1−*S*, up as far as 0.1, making the 1−*S* values accurate to within ±0.0005).

Consider instead a *vector* image containing the same curve, on the same 1.6-in (=72×1.6=115.2 points) vertical scale. Because the co-ordinates given in the PostScript file exported by Adobe Acrobat are recorded to three decimal places, the resolution is *ΔS*=1/(115.2×1,000)≈0.00001, or 200 times that of the raster image.

While both of these resolutions give adequately precise measures of  and allow one to determine how many events are involved in each jump, they may not give such precise measures of the number at risk at each jump, since it is measured as the *reciprocal* of . As an empirical assessment of the precision of the derived measurements, Figure 2 shows the estimated numbers from a raster image and a vector image, along with - as a validity check - the reported numbers at risk at the end of each time interval. They match very well with those given in the articles.

The accuracy can also be quantified using a theoretical error analysis. Consider two adjacent values on the same cumulative incidence curve, where the vertical axis goes from 0% to 5%, reported (after some rounding) to be *y*_0_ and *y*_5_ points, respectively, above some landmark; suppose that without rounding, they would be *Y*_0_ and *Y*_5_ points above. Denote the vertical locations (similarly rounded) of the two adjacent points on the graph as *y*^′^ and *y*^′′^, with *y*^′′^>*y*^′^, corresponding to unrounded values of *Y*^′^ and *Y*^′′^. Then, the estimates of the number at risk is as follows:


In the Appendix, we provide the variance of this derived quantity, assuming that the errors (*e*s) contained in the four *y*s are equal and independent of each other. In practice, the PostScript points are rounded to three decimal places; thus, the true location *Y* associated with a reported location of *y*=563.384 points lies between 563.3835 and 563.3845 points. If errors are uniform over this 0.001 range such that  points, then the coefficient of variation (CV) is


Similarly, if points are rounded to two decimal places, then the corresponding CV is 0.84% [1].

## Results

### Example

Figure 2 refers to a study by Pearson and colleagues [18]. With nonvalvular atrial fibrillation but high risk for stroke, 14,264 patients were randomly assigned to receive either warfarin or rivaroxaban. The investigators sought to determine whether rivaroxaban was non-inferior to warfarin for the primary end point of stroke or systemic embolism. The published cumulative event rates are shown in the left panel of Figure 2. We processed this image by applying our R function to the PostScript file. The right panel in Figure 2 shows the highly accurate estimates of the {*n*_*j*_} provided by PostScript data alone. The numbers were derived by applying Equation 1 to the *S*(*t*_*j*_) estimates derived from the PostScript commands. The numbers at risk at days 0, 120, and 840, were reported at the bottom of the figure in the article. Clearly, even if they had not not provided, they could have been very accurately estimated just from the successive *S*(*t*_*j*_) estimates alone (the slight lack of monotonicity in series (a) in Figure 2 reflects rounding errors in the PostScript co-ordinates). Moreover, the successive *S*(*t*_*j*_) estimates provide accuracy estimated of the numbers at risk at not just at this limited number of time points but also at all time points at which there was at least one event. The slight lack of monotonicity in series (a) reflects rounding errors in the PostScript co-ordinates. Each *n*_*j*_ in series (b) is based on the (clearly false) assumption that the corresponding *d*_*j*_=1; at these distinct failure times, clearly, *d*_*j*_=2, so each *n*_*j*_ is twice that shown. Likewise, the *n*_*j*_s in series (c) are based on assuming *d*_*j*_=1, when, again clearly, *d*_*j*_=3, and the *n*_*j*_ should be three times that shown. This also shows how a *d*_*j*_=1 can be reliably distinguished from a *d*_*j*_=2 or *d*_*j*_=3 simply by inspection.

### An unexpected data disclosure bonus

Originally, to extract the ERSPC [19] data, Hanley used Acrobat Reader to zoom in on the figure so that it filled the screen. He pasted a screenshot of this into the GraphClick software to digitize the two curves. From these, and interpolated numbers at risk for years 1 to 4, 6, 8, and 9 and imputed numbers at risk for years 11 and 12, he was able to compute the estimated yearly numbers of deaths and man-years at risk.

In his subsequent pursuit for greater precision, he noticed that when the figure in the ERSPC report is enlarged in Acrobat Reader, the re-drawing takes a surprisingly long time. Even though the total sample size was 162,000 men, there were only 540 deaths, and so, allowing for some multiplicities, there should be even fewer than that many steps in the two-step functions. Curiosity prompted him to convert the PDF file to PostScript and examine how the steps were drawn. To his surprise (and the disbelief of the study epidemiologist who has told him that the curves had been computed and drawn using Stata but that it was impossible from what was in the figure to go back from them to what he had requested), the PostScript file contained the exact coordinates of each of 89,308 and 72,837 line segments or dots, one per man! This explained why the curves took so long to be re-rendered by Adobe Reader and the page to be printed. The horizontal and vertical coordinates of each of these segments/dots thus provided the exact numbers of men being followed at each point in follow-up time and thus at the exact times of the vertical steps in the curves (corresponding to prostate cancer deaths). The number of prostate cancer deaths at each time point was obtained by multiplying the size of the step by the number being followed at that time. The numbers were then aggregated by year and study arm to produce the counts listed in Figure 1b in the published re-analysis [3].

**Figure 3:**
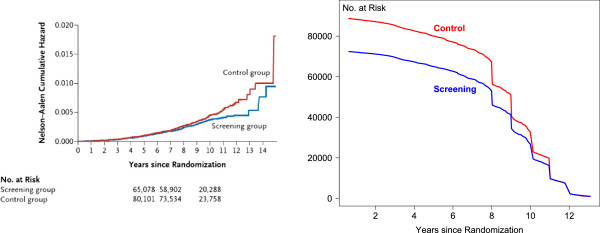
**Nelson-Aalen curves and estimated numbers at risk at each time point.** (Left) Screenshot of the Nelson-Aalen curves in the original NEJM report of the ERSPC and (right) numbers at risk at each time point after randomization, derived from the PostScript file. The large numbers censored exactly at the end of follow-up years 8, 9, 10, and 11 are because the men in the Finnish portion of the trial were randomized on January 1, 1996, 1997, 1998, and 1999 and were still alive on December 31, 2006. The shallower slope of the curve in years 1 to 8 is due to deaths, while the steeper slope of the curve in years 9 to 13 reflects the staggered entries, beginning in different years in the seven different countries.

To illustrate *just how much* data are disclosed by the way Stata makes the curves, we present side by side in Figure 3 the original NEJM figure on the left, together with on the right the ‘numbers of men at risk’ curves that we were able to recreate using the data contained in the PostScript file ‘behind’ the figure on the left. The unusual shape of each ‘numbers at risk’ curve - which we derived from the PostScript data behind the published figure - is explained by the recruitment method. In the ‘Methods’ section of the NEJM article, we read that, in the Finnish portion of the study,

men were recruited at the ages of 55, 59, 63, and 67 years. (...) the size of the screening group was fixed at 32,000 subjects. Because the whole birth cohort underwent randomization, this led to a ratio, for the screening group to the control group, of approximately 1:1.5. (...) Follow-up for mortality analyses began at randomization [January 1 in each of 1996, 1997, 1998 and 1999] and ended at death, emigration, or a uniform censoring date (December 31, 2006).

The 160,00 data points in the Kaplan-Meier curves in the ERPSC report were produced by an early version of Stata. To test whether the latest version continues to draw each censored observation as an invisible dot on the curve, we used Stata version 12 to construct a Kaplan-Meier curve based on the same AML data we used in Figure 1 and to save it as a PDF file. We then used Adobe Acrobat to export it to a PostScript file and extracted the line segments (the .pdf, .ps, and .R files are provided on the website). They reveal that the Stata curve was drawn using 20 line segments - 1 for each of the 7 vertical steps, 1 for each of the 6 horizontal lines for the intervals that do not contain a censored observation, 2 each for the 2 horizontal lines, 2 for the 2 intervals that contain 1 censored observation each, and 3 for the 3 censored observations that do not coincide with a vertical step.

### Distortions produced by further processing

Interestingly, in the ERSPC figure, while the numbers and sizes of the jumps do make sense, the numbers at risk, derived by simply counting how many observations (each one plotted as a dot) exceed the time point in question, do not agree perfectly with those would have obtained from the successive survival ratios described in the ‘Principles’ section above. We traced this discrepancy to the fact that, even when just one death is involved, the jumps implied by the PostScript data are not entirely monotonic, suggesting either some rounding at the time they were generated in Stata, or some post-Stata processing by other graphics software. Given the very large numbers at risk, and thus the very close agreement between the two, the fact that they are Nelson-Aalen rather than Kaplan-Meier curves does not explain the discrepancies. This post-processing seems to be common and sometimes results in quite elaborate ways to draw what appear to the eye as simple step functions. In the exemestane for breast cancer study [20], for example, it took almost 2,500 line segments to produce two-step functions based on a total of 43 events!

## Discussion

The availability of raster-based images, and the practical tools provided by authors such as Tierney et al. [12] and Guyot et al. [4] are particularly valuable in recovering the raw data. As they and now we have shown, one can reliably recover much of the original information that seems to be ‘hidden’ [7] beneath published survival curves.

A digitizer provides more accurate and precise measures of the jumps or ratios. However, the screen itself has limited resolution, and much greater resolution is possible if the original images can be obtained as a PostScript file. The data recovered from a PostScript file can then either be input into these tools or processed directly.

The most time-consuming task in extracting the relevant co-ordinates from a PostScript file is visually searching through the file to find the commands that draw lines or dots and skip the large number of irrelevant commands. We did find that the R package grImport imports PostScript images. Its main focus is adding the extracted images to R graphical plots, but the author’s webpage gives a reference [13] where he describes extracting data from a survival curve and shows that the resulting curve matches the original. The package requires Ghostscript and does not handle the PostScript output produced by more recent versions of Adobe Acrobat. Thus, we wrote our own R function. It does not use intermediate software but extracts the same graphics ‘paths’ as grImport does.

Some PostScript files contain more information that one would need to draw simple step functions. Thus, in some instances, end users may have to do some further processing or select just parts of the overly elaborate paths used to create lines. We have found that some of the graphic files that authors submit with their manuscripts must have been touched or redrawn by the publishers. The Postscript used by Stata seems to disclose considerably more of the data hidden behind survival curves than that generated by other statistical packages that we have explored.

We found many grainy images in some of the best journals and would like to recommend that journals require the submission of device-independent vector-based graphics, such as PDF and EPS figures, rather than raster images to ensure portability and reproducibility.

## Conclusions

When it is not possible to obtain the raw data from the authors, reconstruction techniques are a valuable alternative. Compared with previous approaches, which use manual digitation of raster images, our method takes advantage of the much greater precision of vector-based images rendered via PostScript. The extraction is replicable and avoids the observer variation that accompanies the digitization process.

## Appendix

### Error analysis

If we take two adjacent points on the same cumulative incidence curve and the *y* axis goes from 0% to 5%, then the estimate of the ratio is [20(*c*−*d*)−(*a*−*d*)] /[20(*c*−*d*)−(*b*−*d*)] and thus


where *a* and *b* are the heights of two points on the curve, *c* and *d* are the values corresponding to 5% and 0%, *μ*_1_ and *μ*_2_ are the error-free numerator and denominator, i.e., before any loss of data, and *e*_1_ and *e*_2_ are the errors associated with them, i.e., the observed data with rounding.

Assuming all four error variances are equal to  and independent of each other, then


where , , and covariance .

Further assuming *μ*_1_≈20×100=2,000 points, *μ*_2_≈0.5 points, and , we have


and coefficient of variation


Therefore, if the PostScript points are rounded to three decimal places, then 563.384 points probably lies somewhere (uniformly) between 563.3835 and 563.3845, so error range = 0.001 leads to  and .

Similarly, if the PostScript points are rounded to two decimal places, then 563.38 points probably lies somewhere (uniformly) between 563.375 and 563.385, so error range = 0.01 leads to  and .

### Further examples, elaborated on website

1. Colistin for the treatment of ventilator-associated pneumonia [21]. This report is interesting for two reasons: the fact that despite including this descriptor in the title, it is not a case-control study and the contradictory information in the Kaplan-Meier curve. The correspondence pointed out that the K-M curves seemed to be based only on those who died, but the authors deflected the criticism by noting, correctly, that ‘when two or more events can coexist at a specific time, so the drop can be twice as large or more.’ We leave it to the interested reader to use the JPG files one can export from the PDF file to determine if - as seems to the naked eye - 6 of the jumps in the combination arm in Figure 2 are of size 1/8th each, and 1 is of size 2/8, at variance with the 11 deaths reported in Table 1 of [21], and only possible if all of the 43 - 8 observations were censored before the very first death at day 7 or 8. In this small example, the answers from a digitizer would probably be sufficiently accurate to determine that indeed, the curves seem to be based only on those who died.
2. Marriage risk of cancer research fellows [22]. The Lancet recently attempted to match the whimsical nature of the articles in the Christmas Edition of the BMJ, by publishing a ‘marriage-free survival’ curve in an article. The article began ‘Research fellows aiming to obtain a PhD or MD/PhD degree face many hazards at work, including exposure to toxic substances and harassment by reviewers of their papers’ and lamented the fact that ‘However, few data exist on the sociocultural risk factors encountered at work - eg, their risk of marriage.’ The data and the curve provide a useful teaching example, small enough to be worked by hand, and to have students figure out when and how many ‘individuals with a bachelor status were censored at the time of analysis.’ As can be seen in the correspondence on the website, the authors gladly shared the 13 observations with Hanley, so that teachers can be spared having to reverse engineer them in order to check that their students did so correctly.
3. Rosuvastatin to prevent vascular events in men and women with elevated C-reactive protein (JUPITER) [23]. The report of this study has prompted some concerns about how the number needed to treat was calculated, using 5-year risks that were based on survival curves that ended at year 4.5, but that, because of small numbers of events, were quite erratic in years 4 and 5. The projections also raised the issue of whether (as in our screening example) reductions in event rates are immediate, or delayed, and how long they persist after statins are discontinued. The authors did not answer our request that they share just the half yearly numbers of deaths: we wished to use them, along with the half yearly numbers of at risk that were included in the figures, to calculate time-specific hazards and hazard ratios. Fortunately, even though the placebo and Rosuvastatin curves were displayed in a rectangle less than 60 printer’s points, or 5/6ths of an inch, tall and just over 1 in wide, it was possible to use the PostScript commands to quite accurately determine where along the 4.5-year time axis the unique death times were located and how many there were at each time point.
